# Promotion

**Published:** 1872-03

**Authors:** 


Promotion.—We clip the following from *The Sunday Herald and Weekly National Intelligencer*:

"Assistant Medical Purveyor J. H. Baxter was confirmed by the Senate on Thursday last as Chief medical purveyor, U. S. army. This is a position of great responsibility, as the chief medical purveyor has supervision of all matters pertaining to the purchase of medical and hospital supplies for the army. This tribute to a deserving officer, as well as to the volunteer medical staff of the late war, is very gratifying, as Dr. Baxter was the only one of their number who was selected for a higher position than assistant surgeon in the regular service when the army was reorganized after the close of the war, and his selection for this position is an admirable one. We are glad to learn that his promotion will not take him from among us, as he is still engaged in preparing his report of the medical statistics of the late provost marshal general's bureau, which is near completion."

We congratulate Dr. Baxter on his promotion, and also the service on securing so able a man for the important post of Medical Purveyor.

